# Comparative growth analysis of capsulated (Vi+) and acapsulated (Vi-) Salmonella typhi isolates in human blood

**DOI:** 10.17179/excli2014-674

**Published:** 2015-02-09

**Authors:** Sadia Liaquat, Yasra Sarwar, Aamir Ali, Abdul Haque

**Affiliations:** 1 Enteric Pathogen Laboratory, Health Biotechnology Division, National Institute for Biotechnology and Genetic Engineering (NIBGE), Faisalabad, Pakistan affiliated with Pakistan Institute of Engineering and Applied Sciences (PIEAS), Islamabad, Pakistan; 2 Department of Bioinformatics and Biotechnology, GC University, Faisalabad, Pakistan; 3 Dean, Faculty of Health Sciences, University of Faisalabad, Pakistan

**Keywords:** Salmonella Typhi, Vi+, Vi-, growth, human blood

## Abstract

*Salmonella enterica* serovar Typhi (*S*. Typhi) is a human restricted pathogen. It biosynthesizes a virulence capsular polysaccharide named as Vi antigen. *S*. Typhi regulates expression of genes involved in the biosynthesis of Vi antigen in response to osmolarity. Beside Vi-positive isolates, Vi-negative (acapsulated) isolates are also pathogenic. However, Vi-positive isolates are more prevalent. The present study was planned to investigate comparative growth of Vi-positive and Vi-negative *S*. Typhi isolates in an *ex vivo* human whole blood model. Four isolates of each type were tested for growth in human whole blood and in an enrichment medium (Tryptic soy broth-TSB) as a control. It was found that capsulated (Vi-positive) strains formed smooth circular colonies and grew with shorter lag and generation time than Vi-negative isolates. Overall growth pattern of *S*. Typhi isolates both in vitro and *ex vivo* conditions showed that Vi-positive isolates grew at a faster rate. Especially in human blood, the lag time of acapsulated isolates was almost doubled as compared to capsulated *S*. Typhi isolates. It was also observed that Vi-negative isolates reduced in number up to 81 % during the first 12 hours of incubation in human whole blood. Interestingly, both types of isolates had similar growth curve in TSB indicating that Vi capsule is dispensable for bacterial growth *in vitro*. This study shows for the first time that absence of capsular antigen retards the growth of Vi-negative isolates on initial contact with human blood, but with passage of time they adjust themselves according to the new environment.

## Introduction

*Salmonella enterica* serovar Typhi is an exclusively human pathogen and a leading cause of enteric fever worldwide. It causes an acute, systemic infection of the reticuloendothelial system. Our knowledge of the actual mechanism of pathogenesis of *S*. Typhi is limited because it infects only humans, which means that animal models are not available to study host-pathogen interactions. Most of the current understandings about the pathogenicity of *S*. Typhi have been extrapolated from *S*. Typhimurium infections in mice. A major limitation for using this murine model is that *S*. Typhimurium does not cause typhoid fever in humans but instead causes a localized gastroenteritis resulting in diarrhea. *S*. Typhi produces a virulence factor, the Vi capsular antigen which is absent in *S*. Typhimurium. Vi capsule is synthesized by genes located on a large pathogenecity island designated as SPI-7 which is not present in the genome of *S*. Typhimurium. SPI-7 is genetically unstable (Bueno et al., 2004[3]; Nair et al., 2004[15]) and its deletion may give rise to acapsulated *S.* Typhi isolates. Therefore, on the basis of capsule formation, *S*. Typhi can be grouped into two types: capsulated (Vi-positive) and acapsulated (Vi-negative). Both types have been identified and isolated from typhoid patient’s blood (Baker et al., 2005[1]; Pulickal et al., 2013[16]; Wain et al., 2005[24]) and volunteer studies have shown that both can cause enteric fever (Hone et al., 1988[9]). 

During the course of infection, Vi capsular antigen protects bacteria against host immune system due to its anti-inflammatory (Crawford et al., 2013[4]; Haneda et al., 2009[7]; Raffatellu et al., 2007[17]), antiopsonic and antiphagocytic property (Wilson et al., 2011[27]). The expression of genes involved in the Vi capsular biosynthesis is greatly influenced by the environmental conditions. The osmolarity of the lumen of small intestine is equivalent to 0.3 M NaCl (Sleator et al., 2007[20]) and bacterial exposure to this high osmolarity results in drastic decrease in the expression of Vi antigen (Tartera and Metcalf, 1993[21]; Tran et al., 2010[23]). It is expressed when *S*. Typhi transits from intestinal lumen into the ileal mucosa under low osmolarity environment which may explain the protective role of capsule during systemic infection.

Role of Vi antigen in survival of *S*. Typhi has been studied using macrophage (Sizemore et al., 1997[19]; Wilson et al., 2011[27]), neutrophil (Wangdi et al., 2014[26]) and epithelial cell lines. The role of Vi antigen in serum resistance has also been reported (Bravo et al., 2008[2]; Looney and Steigbigel, 1986[11]). However, during infection, the behavior of capsular and acapsular variants of S. Typhi in whole human blood is still in question. Two conflicting reports have described the role of Vi antigen in protection of *S*. Typhi from inhibitory effect of human serum (Bravo et al., 2008[2]; Looney and Steigbigel, 1986[11]). According to Looney and Steigbigel (1986[11]), Vi-positive strains are more resistant to lysis by non-immune serum than Vi-negative isolates, whereas Bravo et al. (2008[2]) demonstrated that the Vi antigen does not contribute to serum resistance.

This study is based on the comparative growth behavior of Vi-negative and Vi-positive variants of *S*. Typhi in human whole blood as an *ex vivo* model. This comparative study on survival and growth of naturally occurring Vi-negative and Vi-positive *S*. Typhi strains was conducted to investigate whether Vi antigen expression is enhanced within human blood due to low osmolarity conditions, and consequently there are different growth rates of Vi-negative and Vi-positive *S*. Typhi isolates in blood. 

## Materials and Methods

### Bacterial strains

Four Vi-positive and four Vi-negative *S*. Typhi isolates cultured from blood samples of typhoid patients were investigated in this study. Blood samples were collected from different patients suffering from typhoid fever in various hospitals in Faisalabad region of Pakistan. All isolates were revived from National Institute for Biotechnology and Genetic Engineering (NIBGE) stocks stored in 20 % glycerol at -20 °C (Baker et al., 2005[1]).

### In vitro growth analysis

Tryptic soy broth (TSB), a universal culture medium free from inhibitors and indicators with osmolarity (300 mosmol/liter) similar to human blood (Scharfman et al., 1996[18]), was used for reviving the *S*. Typhi stock cultures for *in vitro *growth analysis. Growth in TSB was subcultured on MacConkey agar. A single colony was reinoculated in 3 ml TSB and cultured at 37 °C with shaking at 200 rpm for at least 18 hours. Fifty µl of this overnight culture were inoculated into 3 ml of fresh TSB and cultured at 37 °C with shaking at 200 rpm. At time points of 0, 2, 4, 6, 8, 10, 12, 14, 16, 18 and 24 hours after inoculation, samples of bacterial cultures were collected and used for analysis after making ten fold serial dilution in autoclaved distilled water. The CFU were quantified by plating the appropriate dilutions on MacConkey agar. Each sample was analyzed in triplicate and the analysis was repeated at least three times. The bacterial counts were transformed into logarithms and their average values of CFU/ml were used to plot the growth curve. The generation time was determined by comparing bacterial cell numbers at two time points of the exponential growth phase using following formula (Liu et al., 2006[10]):

Generation time = 0.301 (T_2_ - T_1_) / (logN - logN_0_)

T_1_ is the first time point (minutes) and T_2_ is the second time point of the exponential phase, N_0_ is the number of bacterial cells at T_1_, and N is the number of bacterial cells at T_2_.

### Human whole blood as ex vivo growth model

Human whole blood has been used as an *ex vivo* model for studying the human restricted pathogens including *Neisseria meningitidis*,* Listeria monocytogenes*, and group A and group B *Streptococcus species *(Echenique-Rivera et al., 2011[5]; Graham et al., 2005[6]; Mereghetti et al., 2008[14]; Toledo-Arana et al., 2009[22]). *S*. Typhi is also a human restricted pathogen. Therefore, in this study human blood from healthy volunteers was used as *ex vivo *model for comparative growth analysis of Vi-negative and Vi-positive *S*. Typhi isolates with that of TSB medium (as *in vitro* model). Human venous blood was collected (in heparin) from 4 healthy volunteers (two males and two females, ages ranged from 25 to 35 years) who were not on any antimicrobial treatment for the past eight months before this study. 

Bacterial loads in typhoid patients can reach up to 10^4^ bacteria/ml (Massi et al., 2005[12]; Wain et al., 2001[25]). Therefore, approximately 10^3^ bacteria from early exponential phase culture within TSB were mixed with heparinized blood from each donor separately and cultured at 37 °C with shaking at 200 rpm. To check the *S*. Typhi adaptation in human blood, samples were collected at five different time points (each time point consisted of triplicate culture): time 0 (immediately after mixing bacteria with blood), and after 12, 24, 48 and 72 hours incubation at 37°C with shaking at 200 rpm and used for analysis by serial dilution as in case of *in vitro* growth analysis. Human blood from each donor without bacterial inoculum was used as negative control during viable bacterial cell enumeration experiment.

### Statistical analysis

Two-way ANOVA with Bonferroni posttest was used for the statistical analysis. The statistical analysis was performed using GraphPadPrism5 with the significance set at a P of <0.05. 

## Results

**Figure 1:**
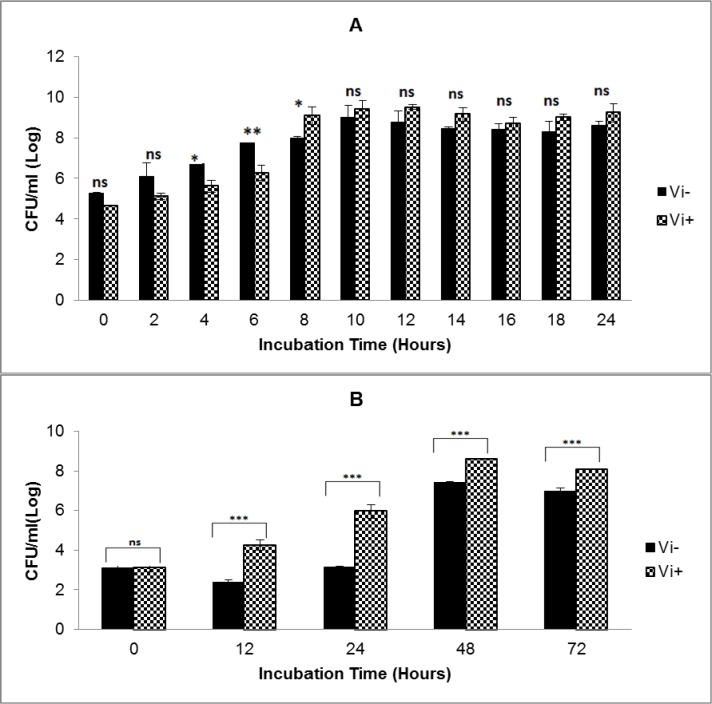
Growth of Vi-negative and Vi-positive *S*. Typhi isolates in TSB medium (A) and human whole blood (B)

**Figure 2:**
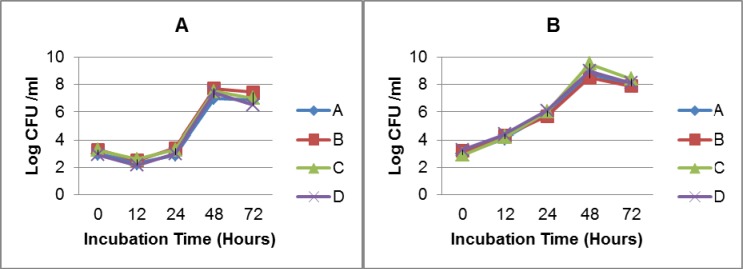
Growth of Vi-negative (A) and Vi-positive (B) *S*. Typhi isolates in human whole blood from four donors

Growth analysis of Vi-negative and Vi-positive *S*. Typhi isolates in TSB medium and human blood from four different donors over time course of 72 hours revealed that Vi-positive isolates have significantly shorter lag and generation times as compared to Vi-negative strains (Table 1(Tab. 1)). Vi-positive isolates grew at much faster rate as compared to Vi-negative isolates. In human whole blood, the lag time of Vi-negative isolates was doubled as compared to Vi-positive isolates. The viable cell density (CFU/ml) of *S*. Typhi capsulated strains (Vi-positive) was increased approximately 14 fold during 12 hours of incubation in blood. Contrasting growth pattern was observed in case of acapsulated isolates (Vi-negative). There was a fivefold (81 %) reduction in numbers after the first 12 hours of incubation in human blood (Figure 1B(Fig. 1)). They also showed different colonial morphology on MacConkey agar. Vi-positive isolates grew in the form of smooth circular colonies whereas Vi-negative isolates had circular colonies with rough boundaries having similar sizes (approximately 2-3 mm within 48 hours of incubation). On the whole, bacterial numbers increased approximately 10^4^ (Vi-negative isolates) and 10^5^ (Vi-positive isolates) over an incubation period of 72 hours in human blood. The bacterial count pattern for Vi-positive isolates was similar in blood of four donors. It was also similar in case of Vi-negative isolates (Figure 2(Fig. 2)) indicating reproducibility of the results.

## Discussion

Vi capsular polysaccharide has been known as a virulence factor of *S*. Typhi for the last several decades. This virulence antigen is expressed by *S*. Typhi, *Citrobacter fruendii*, and some other serogroups of *Salmonellae*. In recent years, the old dogma that virulence of *S*. Typhi is essentially related to production of Vi antigen is shaken with the reports of Vi negative but pathogenic strains emerging from all over the world (Baker et al., 2005[1]; Hone et al., 1988[9]; Mehta and Arya, 2002[13]; Pulickal et al., 2013[16]; Wain et al., 2005[24]). The potential of Vi negative strains to cause typhoid fever is now an established fact but the differences is the growth and survival of Vi negative and Vi positive variants during infection is still not clear. 

The Vi capsule is a protective component of *S*. Typhi and its role in intracellular survival of *S*. Typhi and in serum resistance has been recently reported (Hirose et al., 1997[8]; Looney and Steigbigel, 1986[11]). It is desirable to analyze the effect of absence of this protective antigen during natural infection. The comparative analysis of the growth patterns of Vi-positive and Vi-negative *S*. Typhi isolates in a model mimicking conditions of natural infection can be a method of choice. In this study, human whole blood is selected as an *ex vivo* model because of its ability to mimic the behavior of *S*. Typhi growth in host, in response to cellular and humoral bactericidal mechanisms.

Considering previous reports about serum resistance in the presence of capsular Vi antigen (Bravo et al., 2008[2]; Looney and Steigbigel, 1986[11]), we investigated effect of the presence of Vi antigen on the bacterial survival and growth in human whole blood. Our results showed that presence of capsule affects growth of bacteria in human blood because capsulated isolates grow at a faster rate as compared to acapsulated isolates and they maintain sustainable growth. The lag time of Vi-negative isolates was double as compared to Vi-positive isolates. In case of Vi-negative isolates, live cell density (CFU/ ml) decreased about five fold within the first 12 hours of incubation in blood. It means that acapsulated isolates take longer time to adapt to blood environment where as capsulated isolates promptly acclimatize themselves due to presence of additional protective tool in the form of capsule. This is the major difference in survival and growth of Vi-negative and Vi-positive isolates in human blood as compared to TSB medium (Figure 1A, B(Fig. 1)). Therefore, it may be concluded that absence of capsule affects the survival and growth of Vi-negative isolates in human blood. Additionally, as expected from the analysis of blood from four different volunteers, overall survival pattern of both types of* S*. Typhi isolates remained consistent (Figure 2(Fig. 2)). Thus our results do not agree with a previous study indicating that presence of Vi capsule does not affect bacterial resistance to factors present in human blood (Bravo et al., 2008[2]). Our results showed a marked difference in survival and growth of capsulated and acapsulated isolates in blood within the first 12 hours of incubation.

Looney and Steigbigel (1986[11]) found that Vi-negative *S*. typhi isolates were extremely sensitive to non immune human serum and more than 99 % of bacterial cells were lysed within 40 minutes of their exposure to human serum. Our results support this report, although we found only 81 % reduction in viable Vi-negative bacteria during the first 12 hours of incubation in human blood. Another report by Hirose et al. (1997[8]) pointed out that both types of *S*. Typhi strains can survive in resting macrophages although growth of acapsulated strains was inhibited. Neither Vi-positive nor Vi-negative *S*. Typhi survived in the activated human macrophages and only Vi-positive *S*. Typhi replicated inside resting macrophages as compared to Vi-negative strain (Hirose et al., 1997[8]). Our results support this finding and highlight the protective role of Vi capsule.

In brief, we can say that absence of capsule does not only affect survival but also retard bacterial growth especially during the first 12 hours of incubation in human blood. It is probable that Vi-negative bacteria use some alternative strategies to protect themselves. To best of our knowledge, this is the first report regarding comparative growth analysis of Vi-positive and Vi-negative* S*. Typhi in whole human blood. Thus it can be concluded from the study that the absence of Vi capsular polysaccharide affects the survival of bacteria within human whole blood transiently but can critically affect its growth rate during the course of infection.

## Figures and Tables

**Table 1:**
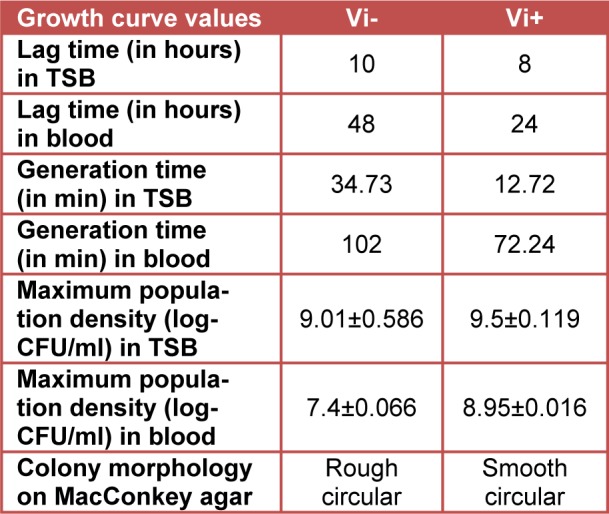
Behavior of Vi-negative and Vi-positive isolates of *Salmonella* Typhi populations incubated at 37 °C in TSB and human blood
